# A trial sequential meta-analysis of *TNF-*α –308G>A (rs800629) gene polymorphism and susceptibility to colorectal cancer

**DOI:** 10.1042/BSR20181052

**Published:** 2019-01-15

**Authors:** Raju K. Mandal, Munawwar Ali Khan, Arif Hussain, Naseem Akhter, Arshad Jawed, Sajad A. Dar, Mohd Wahid, Aditya K. Panda, Mohtashim Lohani, Bhartendu N. Mishra, Shafiul Haque

**Affiliations:** 1 Research and Scientific Studies Unit, College of Nursing and Allied Health Sciences, Jazan University, Jazan-45142, Saudi Arabia; 2 Department of Life and Environmental Sciences, College of Natural and Health Sciences, Zayed University, P.O. Box 19282, Dubai, United Arab Emirates; 3 School of Life Sciences, Manipal Academy of Higher Education, P.O. Box 345050, Dubai, United Arab Emirates; 4 Department of Laboratory Medicine, Faculty of Applied Medical Sciences, Albaha University, Albaha 65431, Saudi Arabia; 5 Centre for Life Sciences, Central University of Jharkhand, Ranchi 835205, Jharkhand, India; 6 Department of Emergency Medical Services, College of Applied Medical Sciences, Jazan University, Jazan 45142, Saudi Arabia; 7 Department of Biotechnology, Institute of Engineering and Technology, Lucknow 226021, Uttar Pradesh, India

**Keywords:** Colorectal cancer, Meta-analysis, polymorphism, susceptibility, Tumor Necrosis Factor-α

## Abstract

Purpose: Tumor necrosis factor-α (TNF-α), secreted by the activated macrophages, may participate in the onset and progression of colorectal cancer (CRC). The association of *TNF*-α **–**308 G>A (rs1800629) single-nucleotide polymorphism (SNP) with CRC risk has been investigated by many studies but the results are inconclusive. A trial sequential meta-analysis was performed for precise estimation of the relationship between *TNF*-α **–**308 G>A gene polymorphism with CRC risk.

Methods: Medline (PubMed), EMBASE (Excerpta-Medica) and Google Scholar were mined for relevant articles. Odds ratios (ORs) and 95% confidence intervals (CIs) were calculated to estimate the significance of association.

Results: The pooled analysis indicated no risk associated with *TNF*-α **–**308 G>A SNP and overall CRC risk in five genetic comparison models, i.e. allelic (A vs. G: *P* = 0.524; OR = 1.074, 95% CI = 0.863–1.335), homozygous (AA vs. GG: *P* = 0.489; OR = 1.227, 95% CI = 0.688–2.188), heterozygous (AG vs. GG: *P* = 0.811; OR = 1.024, 95% CI = 0.843–1.244), dominant (AA+AG vs. GG: *P* = 0.630; OR = 1.055, 95% CI = 0.849–1.311) and recessive (AA vs. AG+GG: *P* = 0.549; OR = 1.181, 95% CI = 0.686–2.033). Subgroup analysis revealed that *TNF*-α **–**308 G>A SNP is associated with reduced risk of CRC in Asian ethnicity. The study showed no publication bias.

Conclusions: No association of *TNF*-α **–**308 G>A SNP with overall CRC risk was found. This SNP is likely to be protective against CRC in Asian population when compared with Caucasian population. Larger prospective-epidemiological studies are warranted to elucidate the roles of *TNF-*α **–**308 G>A SNP in the etiology of CRC and to endorse the present findings.

## Introduction

Approximately 608,000 people lose their life to colorectal cancer (CRC) worldwide [1]. According to the World Health Organization (WHO), millions of people will suffer from symptomatic as well as approximately the equal number from asymptomatic cases of CRC disease in the next decade.

CRC is a very heterogeneous and polygenic disease at a molecular level. It may be the result of interaction among different factors like environmental and genetic [2]. Early genome-wide association studies have shown contribution of many new single-nucleotide polymorphisms (SNPs) to increased CRC risk, showing the involvement of multiple low-penetrance genes in CRC incidence [3]. SNPs may contribute to genomic fragility leading to few critical mutations and eventual CRC onset and progression.

The genetic variants also influence immune response negatively leading to chronic inflammation that may play an important role not only in CRC progression, but also in metastasis and poor prognosis [4]. Therefore, investigation of inflammation-related genetic determinants related to CRC might facilitates the preventive and therapeutic strategies of CRC.

Tumor necrosis factor-α (*TNF*-α) gene consists of four exons with three intervening introns. It is an important pro-inflammatory cytokine secreted by activated macrophages and many other immune regulated cell types like lymphocytes, neutrophils, eosinophils, mast cells and endothelial cells [5].

*TNF*-α also play an important role in apoptosis and angiogenesis by binding to TNFR1 (p55) and TNFR2 (p75) receptor. This binding induces the expression of adhesion molecules, which further facilitate multiple cell signaling cascades that lead to inflammation, invasion and metatstatic tumor cells [6].

The relationship of *TNF*-α associated immune response in the development of CRC is currently a research hotspot [7]. Recent experimental and clinical studies on the role of *TNF*-α have revealed that *TNF*-α plays an important role in the progression of human CRC by inducing epithelial-to-mesenchymal transformation and subsequently assists the invasion and metastasis of CRC [8,9]. Previously published reports suggest that variable production of *TNF*-α is associated with poor prognosis in CRC patients [10]. Moreover, the levels of plasma cytokines including *TNF*-α have been shown to predict the clinical outcomes in patients with advanced CRC [11].

*TNF*-α production is generally regulated at transcriptional level [12]. The polymorphisms located in the promoter region of *TNF*-α gene affects the transcription of *TNF*-α gene. *TNF*-α **–**308 G/A (rs1800629) SNP causing guanine (G) to adenine (A) substitution is located within regulatory hotspot region and thus influences transcription critically. The variant allele A causes loss of transcription factors like activator protein-2 binding, inducing high levels of *TNF*-α when compared with the wild-type allele G [13]. Early reports have shown that this SNP affects cellular function and leads to increased levels of *TNF*-α production [14].

Given the importance of *TNF*-α in CRC development, common functional polymorphism **–**308 G>A of *TNF*-α gene has been studied extensively. However, the results lack consensus among the populations. The association of **–**308 G>A polymorphism of *TNF*-α gene with increased or decreased susceptibility to CRC is still debatable [15–29]. The prime reasons for the inconsistent results among multiple reports may be the different ethnicity of the population along with small sample size in various studies. Low sample size seriously curtails the statistical power required to assess a precise estimate and thus an increase in the sample size may confirm the precise association between *TNF*-α **–**308 G>A gene polymorphism and CRC risk. Therefore, the present study was performed using the already published case–control reports to draw a reliable conclusion on the overall relationship of *TNF*-α **–**308 G>A (rs1800629) gene polymorphism with CRC risk. Meta-analysis is a statistical tool that increases the statistical power and precision in assessment of the effects by using the results of early reports and thus circumventing the issue of small sample size and the insufficient statistical power of individual early genetic studies [30].

## Materials and methods

### Search for relevant literature

An online search was done on different databases like PubMed (Medline), Google Scholar and EMBASE covering all research studies published. The search strings used to retrieve the hits were: Tumor necrosis factor OR tumor necrosis factor-alpha OR TNFA OR *TNF*α OR *TNF*-α OR *TNF* gene (polymorphism OR variant OR mutation) AND colorectal cancer susceptibility OR risk (last updated on February 2018). The relevant studies about genetic association were extracted after perusing their titles and abstracts. The publications suiting the above discussed preset eligibility criteria were considered for further examination. The references of the retrieved reports were also searched for additional relevant reports.

### Criteria for inclusion and exclusion of studies

To keep the heterogeneity in check and right interpretation of the study, following criteria were followed to include the published reports in current meta-analysis: (a) only case–control studies assessing association between *TNF*-α **–**308 G>A gene polymorphism and CRC risk, (b) the study must have recruited clearly defined and confirmed CRC patients and CRC free controls, (d) genotype frequency in cases and the controls should be reported, (e) language of these studies should be English and (f) should have used statistically relevant data collection and analysis methods. Additionally, if the case–control studies derived the cases from the same population, the study having larger number of individuals was selected. Study was excluded based upon: (a) duplicate or overlapping report, (b) report based on only CRC cases, (c) no reported genotype frequency and (d) the data of review or abstract.

### Data extraction

The quality of the data extracted was assessed by two investigators (R.K.M. and M.A.K.) individually following a standard protocol. Preset inclusion/exclusion as well as the sequential exclusion criteria of the unsuitable studies outlined in the data-collection form was strictly adhered to ensure the accuracy of the collected data. Any disagreement between the investigators about the quality of collected data was first subjected to a consensus and then finally settled with an open discussion with the arbitrator (S.H.). The data extracted from the retrieved publications consisted first author name, the country of origin, year of publication, number and source of cases and controls, type of study type, genotype frequencies and association with CRC.

### Quality assessment using Newcastle–Ottawa Scale

Quality assessment of the selected studies was done independently by two investigators, namely A.H. and N.A. This evaluation was done by following the Newcastle–Ottawa Scale (NOS) of quality assessment [31]. The major aspects used for NOS quality assessment criteria were: (a) selection of subjects: 0–4 points, (b) subject comparability: 0–2 points and (c) clinical outcome: 0–3 points. The extracted case–control studies securing 5 or more stars were considered having moderate to good quality [31,32]. In case, if any difference occurred on any item between the above two investigators, the issue was fully discussed and solved by a detailed discussion in the presence of third investigator (S.A.D.) participated as adjudicator.

### Statistical analysis

Pooled ORs and their corresponding 95% CIs were used to appraise the risk association between the *TNF*-α **–**308 G>A gene polymorphism and susceptibility to CRC. Heterogeneity was assessed using the chi-square-based *Q*-test and was considered significant if the *P*-value was less than 0.05 [33]. The collected data from single comparison was calculated using a fixed effects model [34], in case of no heterogeneity. However, the random-effects model [35] was employed for pooling of the data. Further, *I*^2^ statistics used to estimate the interstudy variability in which larger values showed a higher degree of heterogeneity [36]. Hardy–Weinberg equilibrium (HWE) in the controls was calculated using chi-square test. Whereas Egger’s regression test showing the funnel plot asymmetry was used to measure significance of publication bias, if any. Further to this, ethnicity was adopted to perform the subgroup stratified analysis, when data were available. The Comprehensive Meta-Analysis (CMA) software program Version 2.0 from Biostat (NJ), U.S.A. was selected to conduct all the statistical calculations involved in the present meta-analysis.

### Trial sequential analysis

According to the Cochrane handbook, the meta-analyses are acceptable if it includes all the eligible trials. However, it may lack sufficient evidences. The meta-analysis may contain systematic errors (bias) or random errors (play of chance), which can be reduced using novel statistical analysis program named ‘Trial Sequential Analysis’ (TSA) tool, made by Copenhagen Trial Unit, Center for Clinical Intervention Research, Denmark). TSA calculates required information size as well as adjusts the threshold for the statistical significance and finally calculates the robustness of present conclusion [37–39]. Briefly, a TSA monitoring boundary crossed with *Z* curve confirms the presence of robust evidence. In such case further trials are not needed. However, *Z* curve not crossing the monitoring boundaries suggest that the trial should continue. Trial Sequential Analysis (version 0.9, http://www.ctu.dk/tsa/) was used in current study.

## Results

### Literature search

Two investigators (viz*.* R.K.M. and M.A.K.) individually examined every title and abstract of the retrieved studies using the designated online web-databases search in a sequential order. The full-text of each study apposite for the inclusion was also recovered. To evaluate the aptness of the study for the inclusion in this pooled analysis, one researcher (R.K.M.) systematically examined all the full-text retrieved publications. Afterwards, the second researcher (M.A.K.) performed the same procedure of text evaluation independently by selecting randomly 10% of the full-text articles. During the study selection process, complete agreement was found between the above stated two researchers regarding the study exclusion and selection criteria. After the selection of the final set of the eligible studies, another researcher (S.A.D.) extrapolated the pertinent data from all the included studies. This step of data extrapolation was cross-checked by a fourth researcher (A.J.) independently by collecting the information from all the selected articles. Discrepancies and discords occurred during the study selection were resolved amicably with thorough discussion before the adjudicators (S.H. and B.N.M.).

### Properties of the reports included in the present study

**Figure 1:**
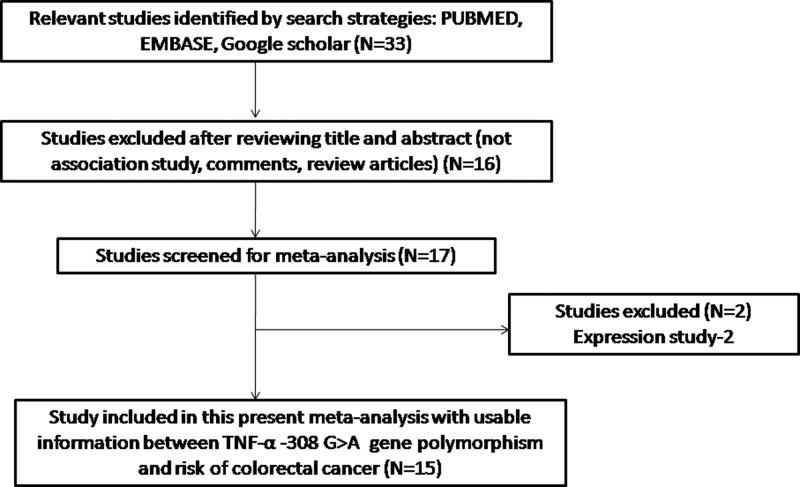
PRISMA 2009 flow diagram depicting identification and selection process (inclusion/exclusion) of the germane published articles dealing with *TNF*-α –308 G>A gene polymorphism and colorectal cancer (CRC) risk for the present meta-analysis

Fifteen articles were selected after systematic literature search done on PubMed, EMBASE and Google Scholar. The retrieved texts were scrutinized by perusing complete texts for their potential relevance for the current meta-analysis (Figure 1). Reports showing *TNF*-α gene polymorphism to estimate survival in CRC patients or using CRC variants as indicators for prognosis were excluded at the onset. Likewise, the reports analyzing TNF mRNA levels or subsequent protein expression were also excluded. The studies with case–control or cohort that design only reporting frequency of all the three genotypes were included for the current meta-analysis. Additionally, all the references cited in the retrieved articles were also scanned to identify other potential case–control studies. Finally, the 15 original publications were found eligible after applying the stringent inclusion and exclusion criteria (Table 1). The genotypes distribution, *P*-values of HWE and susceptibility towards colorectal risk have been given in Table 2. The selected studies (15 in number) were examined for the overall quality following the NOS. Maximum number of the studies included (>80%) scored 5 stars or more, showing a modest to decent quality (Table 3).

**Table 1:** Main characteristics of all studies included in the present meta-analysis

First authors and year	Country	Ethnicity	Control	Cases	Study	Methods	Association
Cho et al., 2017	Korea	Asian	1445	695	HB	Mass array	No risk
Gutiérrez et al., 2016	Mexico	Caucasian	209	164	PB	PCR-RFLP	No risk
Banday et al., 2016	India	Asian	184	142	HB	PCR-RFLP	No risk
Hamadien et al., 2016	Saudi	Asian	100	100	HB	TaqMan	Yes
Stanilov et al., 2014	Bulgaria	Caucasian	177	119	HB	PCR-RFLP	No
Li et al., 2011	China	Asian	180	180	HB	PCR-RFLP	Yes
Tsilidis et al., 2009	USA	Caucasian	372	204	PB	Taqman	No
Garrity et al., 2008	USA	Caucasian	114	114	HB	Sequencing	Yes
Toth et al., 2007	Hungary	Caucasian	141	183	HB	PCR-RFLP	No
Gunter et al., 2006	USA	Caucasian	202	217	HB	Taqman	No
Theodoropoulos et al., 2006	Greece	Caucasian	200	222	HB	PCR-RFLP	No
Macarthur et al., 2005	Scotland	Caucasian	389	246	PB	Taqman	No
Landi et al., 2003	Spain	Caucasian	326	377	HB	Sequencing	No
Jang et al., 2001	Korea	Asian	92	27	HB	PCR-RFLP	No
Park et al., 1998	Korea	Asian	328	140	HB	PCR-RFLP	No

Abbreviations: HB, hospital based; PB, population based.

**Table 2:** Genotypic distribution of *TNF*-α –308 G>A (rs1800629) gene polymorphism included in this meta-analysis

Authors and year	Controls				Cases				
	Genotype			Minor allele	Genotype			Minor allele	HWE
	GG	GA	AA	MAF	GG	GA	AA	MAF	*P*-value
Cho et al., 2017	1192	203	50	0.104	598	90	7	0.074	0.001
Gutiérrez et al., 2016	180	27	2	0.074	139	21	4	0.088	0.391
Banday et al., 2016	150	34	0	0.092	124	18	0	0.063	0.167
Hamadien et al., 2016	59	23	18	0.295	67	23	10	0.215	0.001
Stanilov et al., 2014	135	40	2	0.124	88	28	3	0.142	0.612
Li et al., 2011	160	19	1	0.058	156	15	9	0.091	0.599
Tsilidis et al., 2009	275	90	7	0.139	146	55	3	0.149	0.908
Garrity et al., 2008	92	20	2	0.105	52	49	13	0.328	0.464
Toth et al., 2007	111	30	0	0.106	132	48	3	0.147	0.157
Gunter et al., 2006	139	57	6	0.170	146	59	12	0.191	0.957
Theodoropoulos et al., 2006	146	44	10	0.16	152	56	14	0.189	0.010
Macarthur et al., 2005	224	145	20	0.237	157	74	15	0.211	0.577
Landi et al., 2003	234	76	10	0.15	278	80	5	0.123	0.219
Jang et al., 2001	85	7	0	0.038	24	3	0	0.055	0.704
Park et al., 1998	252	72	4	0.121	115	24	1	0.092	0.651

Abbreviations: HWE, Hardy–Weinberg equilibrium; MAF, minor allele frequency.

**Table 3:** Quality assessment conducted according to the Newcastle–Ottawa Scale for all the studies included in this meta-analysis

First author and year	Quality indicators		
	Selection	Comparability	Exposure
Cho et al., 2017	***	*	**
Gutiérrez et al., 2016	***	*	**
Banday et al., 2016	***	*	***
Hamadien et al., 2016	**	*	**
Stanilov et al., 2014	***	*	**
Li et al., 2011	**	*	**
Tsilidis et al., 2009	***	**	**
Garrity et al., 2008	**	**	**
Toth et al., 2007	***	*	**
Gunter et al., 2006	***	**	**
Theodoropoulos et al., 2006	**	*	**
Macarthur et al., 2005	***	*	**
Landi et al., 2003	**	**	**
Jang et al., 2001	**	*	**
Park et al., 1998	**	*	**

Note: On assessing the quality of the included studies using the Newcastle–Ottawa Scale, all the studies scored five stars or more which indicates no bias.

### Assessment of publication bias

**Figure 2:**
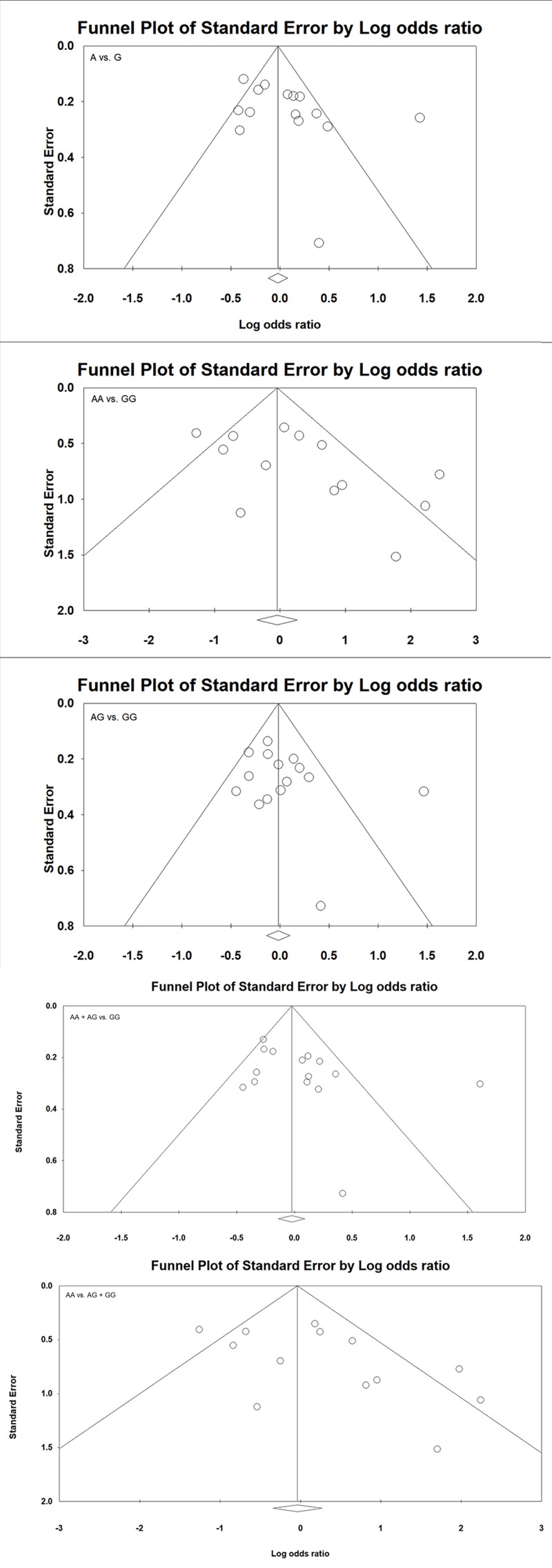
Assessment of publication bias shown with Funnel plots in studies assaying odds of CRC risk associated with the *TNF*-α –308 G>A polymorphism for the overall analysis (odds ratio against standard error in different genetic models)

Begg’s funnel plot and Egger’s test showed no publication bias among all the comparison models (Table 4) in every genetic model and the allelic contrast (Figure 2).

**Table 4:** Statistics to test publication bias and heterogeneity in this meta-analysis of *TNF*-α –308 G>A polymorphism and CRC risk: overall

Comparisons	Egger’s regression analysis			Heterogeneity analysis			Model used for the meta-analysis
	Intercept	95% confidence interval	*P*-value	*Q*-value	*P*_heterogeneity_	I^2^ (%)	
A vs. G	2.67	−0.49 to 5.84	0.09	58.67	0.01	76.14	Random
AA vs. GG	2.24	−0.19 to 4.69	0.07	34.99	0.01	65.71	Random
AG vs. GG	1.35	−1.20 to 3.89	0.27	32.60	0.01	57.06	Random
AA+AG vs. GG	2.21	−0.66 to 5.09	0.12	45.27	0.01	69.07	Random
AA vs. AG+GG	2.02	−0.29 to 4.35	0.08	31.27	0.01	61.63	Random

### Test of heterogeneity

The chi-squared-based *Q*-test and *I*^2^ statistics showed the substantial amount of heterogeneity in all the genetic models leading to the use of random-effects model to process the data (Table 4).

### Quantitative synthesis

**Figure 3:**
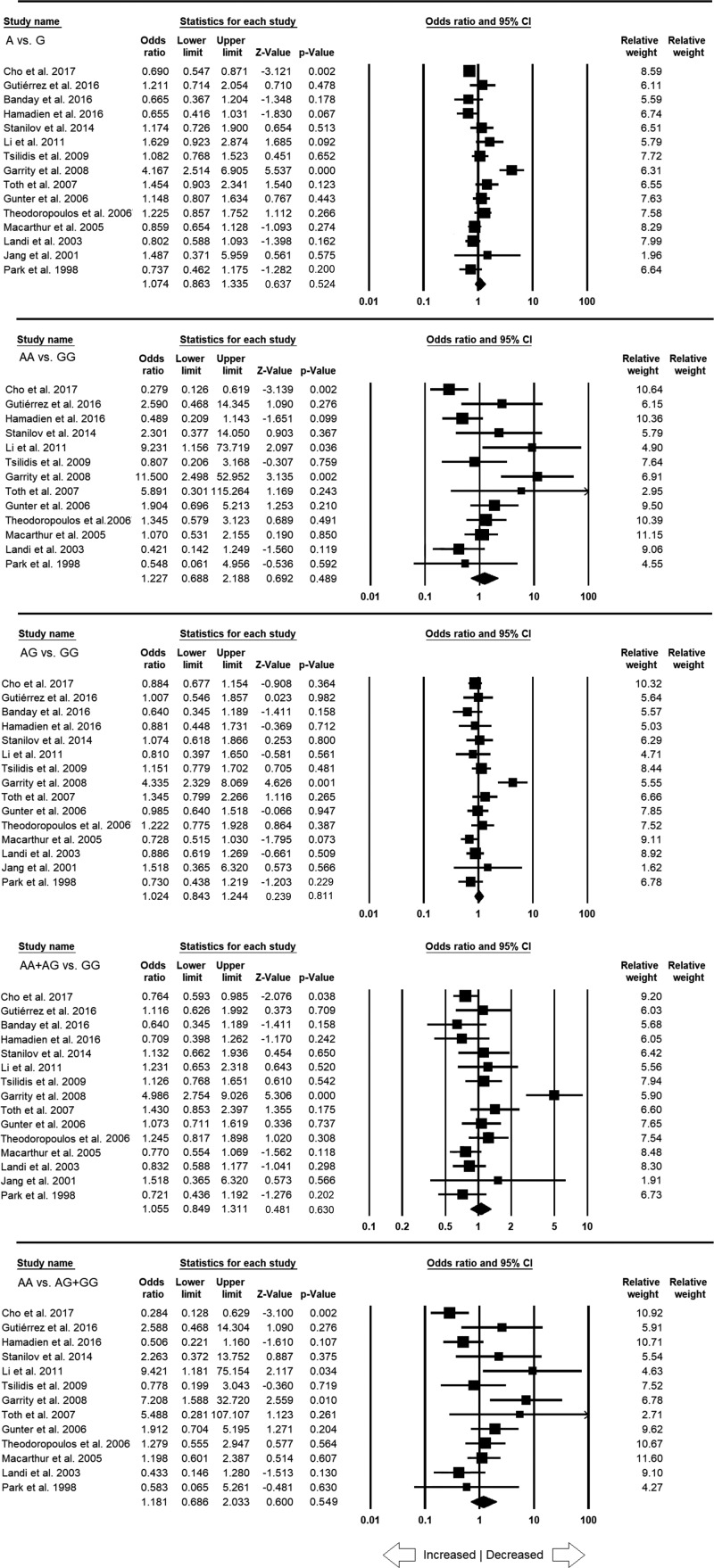
Forest plot of ORs with 95% CI of CRC risk associated with the *TNF*-α –308 G>A gene polymorphism for the overall population Note: Black square represents the value of OR and the size of the square indicates the inverse proportion relative to its variance. Horizontal line is the 95% CI of OR.

All the 15 studies pooled together amounted to 3116 confirmed CRC cases and 4480 healthy controls for the evaluation of overall association between the *TNF*-α **–**308 G>A SNP and CRC risk. The overall ORs showed no statistically significant association with high or low risk between *TNF*-α **–**308 G>A gene polymorphism and CRC risk in neither genetic models (A vs. G: *P* = 0.524; OR = 1.074, 95% CI = 0.863–1.335), homozygous (AA vs. GG: *P* = 0.489; OR = 1.227, 95% CI = 0.688–2.188), heterozygous (AG vs. GG: *P* = 0.811; OR = 1.024, 95% CI = 0.843–1.244), dominant (AA+AG vs. GG: *P* = 0.630; OR = 1.055, 95% CI = 0.849–1.311) and recessive (AA vs. AG+GG: *P* = 0.549; OR = 1.181, 95% CI = 0.686–2.033) genetic models (Figure 3).

### Sensitivity analysis

**Figure 4:**
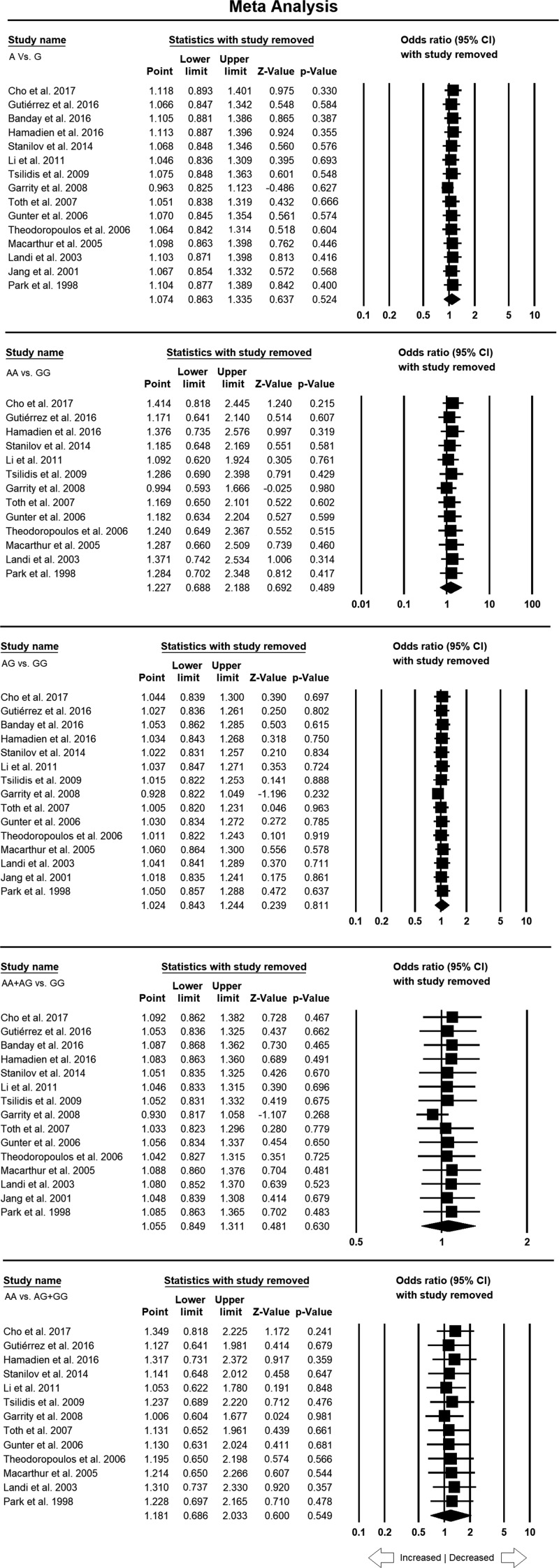
Sensitivity analysis of *TNF*-α –308 G>A polymorphism with overall CRC risk to evaluate the influence of each individual study on the pooled OR by deleting one single study each time for the overall analysis (for all the genetic models) Note: Black square represents the value of OR and the size of the square indicates the inverse proportion relative to its variance. Horizontal line is the 95% CI of OR.

Sensitivity analysis used to evaluate the impact of each individual study on the pooled ORs revealed no significant influence on the pooled OR by any individual study (Figure 4).

### Subgroup analysis: association of the *TNF*-α –308 G>A SNP and risk of CRC in Caucasian and Asian population

A stratified subgroup analysis based on the ethnicity of the enrolled subjects was performed to explore the effect of ethnicity (Caucasian and Asian) on the association of *TNF*-α **–**308 G>A SNP and the risk of CRC onset.

### Subgroup analysis of Caucasian population

**Figure 5:**
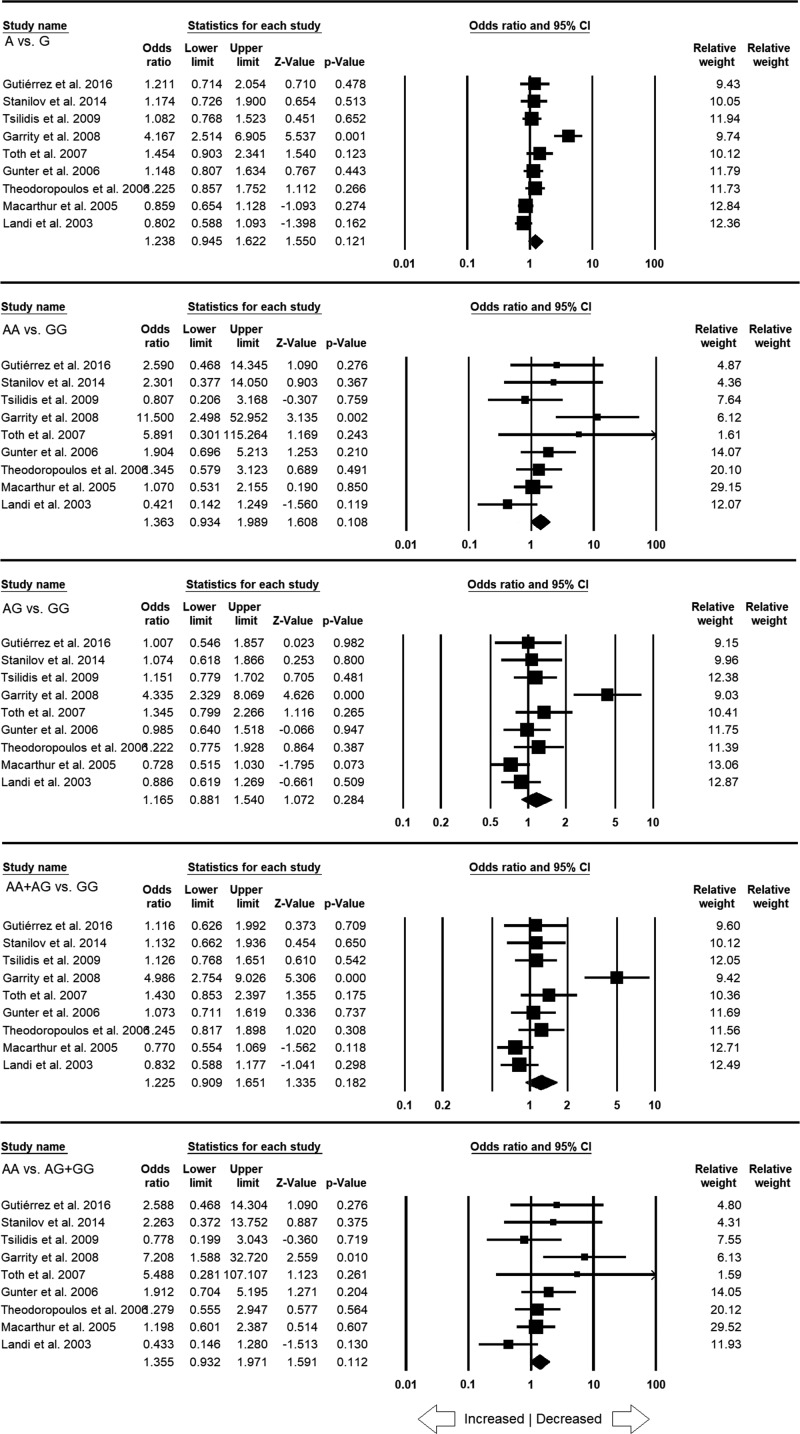
Forest plot of ORs with 95% CI of CRC risk associated with the *TNF*-α –308 G>A gene polymorphism for the Caucasian population Note: Black square represents the value of OR and the size of the square indicates the inverse proportion relative to its variance. Horizontal line is the 95% CI of OR.

Nine case–control studies contain 2130 controls and 1846 cases. These controls and cases were included for subgroup analysis of Caucasian population. The analysis showed significant heterogeneity in three genetic models (Table 5) (Supplementary Figure S1). The conducted analyses using random and fixed models observed no significant association of CRC susceptibility in all the genetic models, i.e. allele model (A vs. G: *P* = 0.121; OR = 1.238, 95% CI = 0.945–1.622), homozygous model (AA vs. GG: *P* = 0.108; OR = 1.363, 95% CI = 0.934–1.989), heterozygous model (AG vs. GG: *P* = 0.284; OR = 1.165, 95% CI = 0.881–1.540), dominant model (AA+AG vs. GG: *P* = 0.182; OR = 1.225, 95% CI = 0.909–1.651) and recessive model (AA vs. AG+GG: *P* = 0.112; OR = 1.355, 95% CI = 0.932–1.971) (Figure 5). Results of the sensitivity analysis are shown as Supplementary Figure S2.

**Table 5:** Statistics to test publication bias and heterogeneity in this meta-analysis of *TNF*-α –308 G>A polymorphism and CRC risk: Caucasian ethnicity population

Comparisons	Egger’s regression analysis			Heterogeneity analysis			Model used for the meta-analysis
	Intercept	95% confidence interval	*P*-value	*Q*-value	*P*_heterogeneity_	I^2^ (%)	
A vs. G	6.43	0.80 to 12.06	0.03	35.67	0.01	77.57	Random
AA vs. GG	1.71	−1.06 to 4.49	0.18	15.22	0.06	47.43	Fixed
AG vs. GG	5.86	0.38 to 11.33	0.04	26.65	0.01	69.98	Random
AA+AG vs. GG	6.93	1.23 to 12.63	0.02	33.29	0.01	75.97	Random
AA vs. AG+GG	1.39	−1.09 to 3.87	0.22	11.89	0.16	32.69	Fixed

### Subgroup analysis of Asian population

**Figure 6:**
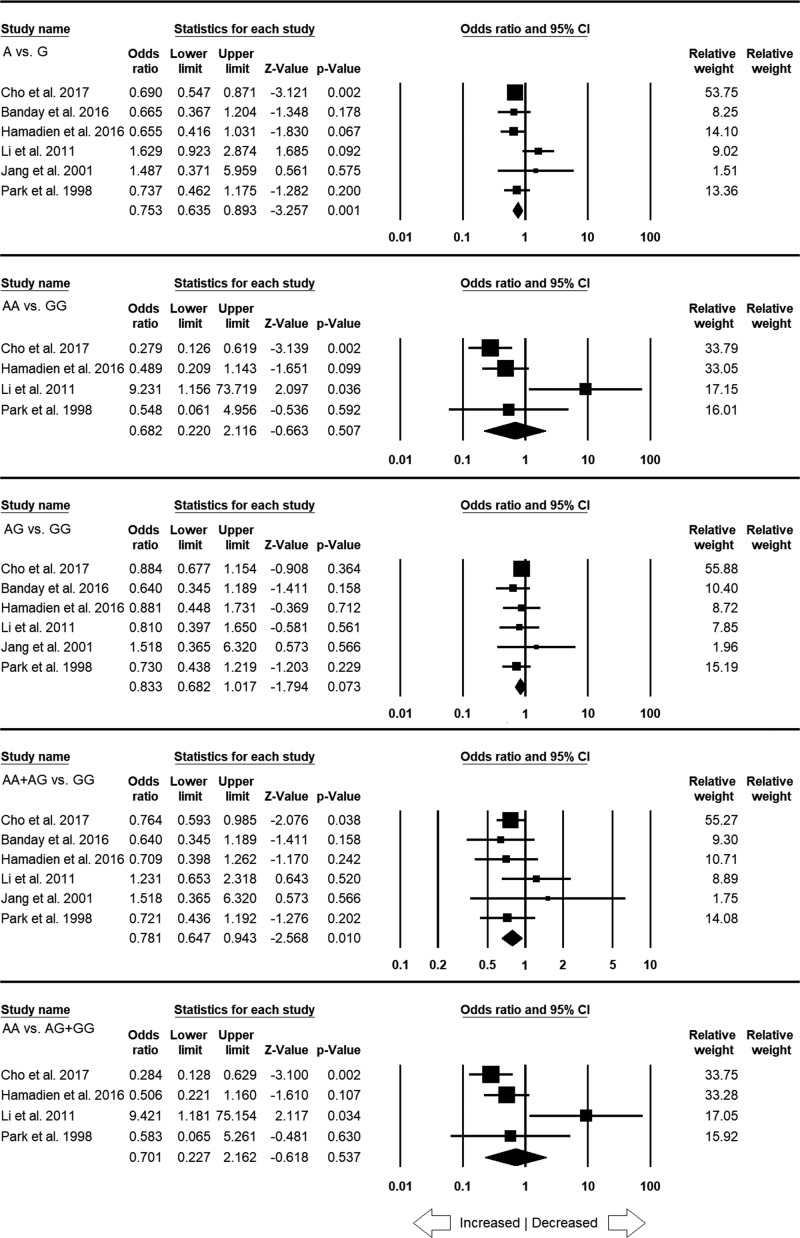
Forest plot of ORs with 95% CI of CRC risk associated with the *TNF*-α –308 G>A gene polymorphism for the Asian population Note: Black square represents the value of OR and the size of the square indicates the inverse proportion relative to its variance. Horizontal line is the 95% CI of OR.

Like Caucasian population, six studies having 2329 controls and 1284 cases were included in the subgroup analysis of Asian population. The analysis showed no publication bias but heterogeneity was observed in two genetic models (Table 6) (Supplementary Figure S3). Interestingly, the protective association of CRC risk with allelic contrast (A vs. G: *P* = 0.001; OR = 0.753, 95% CI = 0.635–0.893) and dominant genetic model (AA+AG vs. GG: *P* = 0.010; OR = 0.781, 95% CI = 0.647–0.943). The remaining three genetic models, i.e. homozygous (AA vs. GG: *P* = 0.507; OR = 0.682, 95% CI = 0.220–2.116), heterozygous (AG vs. GG: *P* = 0.073; OR = 0.833, 95% CI = 0.682–1.017) and recessive (AA vs. AG+GG: *P* = 0.537; OR = 0.701, 95% CI = 0.227–2.162) genetic models showed no link with high or low risk of CRC (Figure 6). Sensitivity analysis results are supplied as the Supplementary Figure S4.

**Table 6:** Statistics to test publication bias and heterogeneity in this meta-analysis of *TNF*-α –308 G>A polymorphism and CRC risk: Asian ethnicity population

Comparisons	Egger’s regression analysis			Heterogeneity analysis			Model used for the meta-analysis
	Intercept	95% Confidence Interval	p-value	Q-value	P_heterogeneity_	I^2^ (%)	
A vs. G	1.45	−1.75 to 4.65	0.28	9.09	0.10	45.04	Fixed
AA vs. GG	2.77	−5.10 to 10.65	0.27	9.56	0.02	68.61	Random
AG vs. GG	0.00	−1.67 to 1.67	0.99	1.85	0.87	0.01	Fixed
AA+AG vs. GG	0.71	−1.38 to 2.80	0.39	3.45	0.63	0.01	Fixed
AA vs. AG+GG	2.76	−5.10 to 10.62	0.27	9.61	0.02	68.79	Random

### Trial sequential analysis of *TNF*-α –308 G>A SNP and risk of CRC

**Figure 7:**
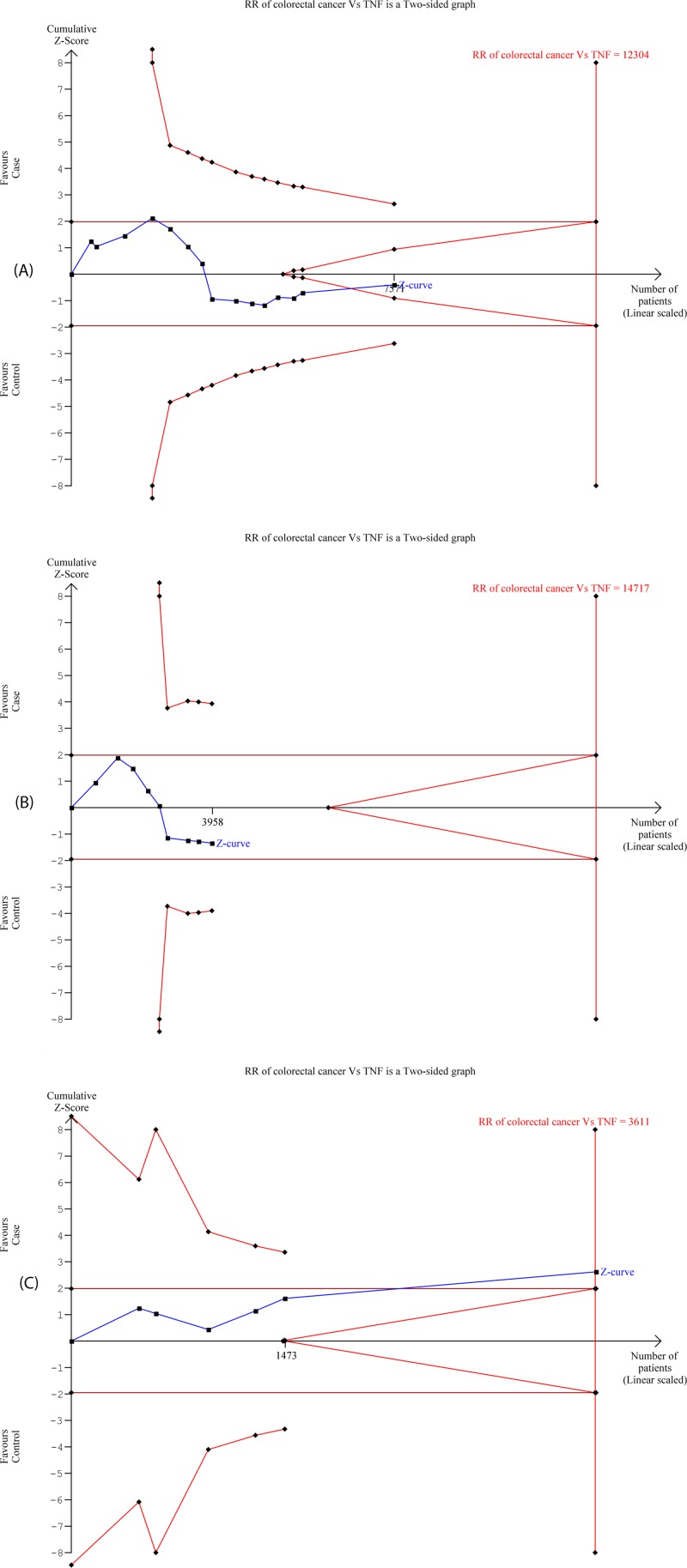
Trial sequence analysis of all the included studies dealing with *TNF*-α –308 G>A gene polymorphisms based on dominant genetic model for (A) Overall, (B) Caucasian, and (C) Asian ethnicity population and CRC risk

TSA (taking the data of the dominant model) was used for the current analysis to check if further trials are required (Figure 7A). Same result was observed after the subgroup analysis based on the Caucasian (Figure 7B) and Asian (Figure 7C) population.

## Discussion

The SNP analysis is useful in genomic DNA screening. It is specifically important in case of CRC because these markers are not affected by disease activity and remain unchanged over time. Research probing genetic associations are powerful methods for identifying low penetrance susceptibility genes that can affect biological process and provide linkage analysis when investigating complex disease like CRC. Translating this information into routinely applied diagnostics would assist in better understanding of the CRC etiology, possibly leading to novel and better clinical practice with many benefits for the patient.

Inflammatory response harmonizes host response against infection participating in repair of tissue, in case of tissue damage. A chronic inflammation many a time alters the immune system and leads to carcinogenesis [40]. Over the time, the cytokines are receiving overdue attention due to their property of mediating and regulating the immune response including inflammation. The cytokines may regulate the pro-inflammatory and anti-inflammatory network to stimulate signaling pathways involved in malignancy development [41].

CRC onset and progression is linked with innate immune processes and inflammation in intestine. Pro- inflammatory genes have key role in maintenance and growth of CRC [42]. *TNF*-α is a pro-inflammatory cytokine made by activated immune cells leading to suppression of tumor proliferation [43]. *TNF*-α also activates antitumor the natural killer cells and CD8 T cells [44].

CRC onset and tumor progression are preceded by inflammation. This has developed a curiosity in scientific community to understand the molecular signaling pathways that connect TNF-α with the development and survival of CRC tumor cells. The expression of *TNF*-α like other cytokines is tightly regulated at the transcriptional level and also at post-transcriptional level. The **–**308 G>A SNP is located inside the regulatory regions of *TNF*-α gene. *TNF*-α expression and secretion both are influenced by this polymorphism.

Many scientists have published their works on CRC, but the molecular and biological mechanism of relationship between *TNF*-α gene polymorphism and risk of CRC is not completely understood. Till date, the reports of *TNF*-α (–308 G>A) SNP in relation with CRC risk lack consensus. Many clinical case–control studies have reported both positive as well as negative association of CRC and *TNF*-α (–308 G>A) SNP. Hence, larger sample size with pooled and subgroup analysis is demand of the time to evaluate the potential role of *TNF*-α –308 G>A polymorphism as a genetic risk factor for CRC infection. The combined ORs from many early reports that lead to large sample size with required statistical robustness also lower the random errors [45]. The meta-analyses address a wide variety of clinical problems using early published data. This meta-analysis included 15 eligible case–control studies comprising 3116 cases and 4480 healthy controls and analyzed the pooled ORs and *P*-value to appraise the precise relationship between the *TNF*-α –308 G>A SNP and CRC risk. NOS quality assessment showed nearly every study scoring five or more than five stars suggesting good to moderate quality of extracted data. The current meta-analysis shows no link between the *TNF*-α –308 G>A SNP and CRC susceptibility by any genetic model in overall population analysis. The speculations based upon the findings tell that numerous polymorphic sites present in the promoter and the coding regions of *TNF*-α gene might serve to keep this gene under tight control and influence the expression of *TNF*-α. Hence, the haplotype combinations might be conserved in certain population to protect against pathogens. Furthermore, the gene reporter assay also testified that A allele of –308 polymorphism does not influence *TNF*-α gene transcription [46]. Many early reports show that –308 G>A polymorphism leads to different transcription rate in *TNF*-α production [47,48].

A stratification analysis of ethnicity was performed considering the fact that polymorphism frequencies might differ among ethnic groups. The separate race-specific meta-analysis done in Caucasian and Asian populations show that *TNF*-α –308G>A SNP is protective against the CRC risk in Asian but not in Caucasians population. The increased plasma concentration of *TNF*-α, which is a result of single *TNF*-α –308 A allele, might not influence CRC risk. The most likely reason might be the population stratification within involved studies, especially when both allelic frequencies and incidence of disease vary across ethnic groups. The range of A allele frequency is from 2 to 9% in Asians, 8 to 10% in South Americans and 10 to 23% in Europeans.

An early meta-analysis by Min et al. showed increased risk of CRC [49]. But, they found increased risk with only homozygous model and less significant risk under heterozygote model in overall population. Furthermore, they have not provided the frequencies of each genotype of included studies and any risk involved in the subgroup analysis. After applying the stern inclusion criteria, the current meta-analysis was added new studies that led to increase in the number of included subjects in both CRC and controls. The analysis was also stratified along race namely Asian and Caucasian. The results of the current analysis tend to be more precise in estimating the relationship between the *TNF*-α –308 G>A SNP and risk of CRC in overall population as well as ethnicity than previous ones.

The previous findings suggested that susceptibility towards CRC is polygenic in nature that indicates the possibility that many genes are participate in determining the resistance or susceptibility to CRC. Consequently, the complex nature of CRC and multifaceted nature of the immune system, *TNF*-α –308 G>A SNP is not the sole reason for the predisposition to CRC, but this polymorphism may interact with other polymorphisms present in linkage disequilibrium of this gene to cause risk.

Despite being many advantages of the present aforementioned study, some limitations must also be mentioned, namely: first, interstudy heterogeneity was observed in the overall comparison from each genetic model. We minimized the likelihood of this problem by performing data analysis by using random-effects model. When studies were stratified by ethnicity, low heterogeneity was found in both Caucasian and Asian populations. Hence, we considered that the racial differences and ethnic origin of the study population, inadequate selection criteria of the subjects, and small sample size of each included study might be responsible for the foremost source of heterogeneity. Second, the present study included the reports published in the English language only. Further, PubMed-Medline, EMBASE and Google Scholar electronic databases were used for the study and subsequent data retrieval. It is possible that some relevant studies published in language other than English or indexed on different databases, are not included. Third, the present study did not have any information on gene–environment interactions due to inadequate data available on this matter. Fourth, the calculations used unadjusted assessment of ORs, which may influence the results.

Despite above limitations, the present study does have many strengths: First, it has included large number of subjects that gave powerful evidence to reach on precise and robust conclusion. It would greatly improve the understanding on the role *TNF*-α –308 G>A SNP in CRC pathogenesis. Second, absence of publication bias and sensitivity analysis suggests the reliability of the results and whole study. Furthermore, all the included studies were of good to modest quality to fulfill the preset needful criteria required by NOS quality assessment.

## Conclusions

The current meta-analysis indicates that *TNF*-α –308 G>A SNP has no role in CRC progression. It also suggests that individuals with *TNF*-α –308 G>A genetic variant have comparatively less CRC risk among Asians. It is a suggestive limited piece of evidence that –308 A allele might reduce CRC risk. As, *TNF*-α plays a significant role in immune response, further larger case–control studies are warranted to make the conclusions more comprehensive. Taken as a whole, the present study would greatly help the scientists in understanding the relationship of *TNF*-α –308 G>A SNP and CRC risk across the world.

## Supporting information

**Figure S1:** 

**Figure S2:** 

**Figure S3:** 

**Figure S4:** 

## Abbreviations

CI: confidence interval
CRC: colorectal cancer
HWE: Hardy–Weinberg Equilibrium
LD: linkage disequilibrium
NOS: Newcastle–Ottawa Scale
OR: odds ratio
SNP: single-nucleotide polymorphism
TNF: tumor necrosis factor
