# Supplementary figures and images for: A trial sequential meta-analysis of TNF-α –308G>A (rs800629) gene polymorphism and susceptibility to colorectal cancer
Source: Biosci Rep. 2019 Jan 15;39(1):BSR20181052. doi: 10.1042/BSR20181052 (PMC6331670; doi:10.1042/BSR20181052)

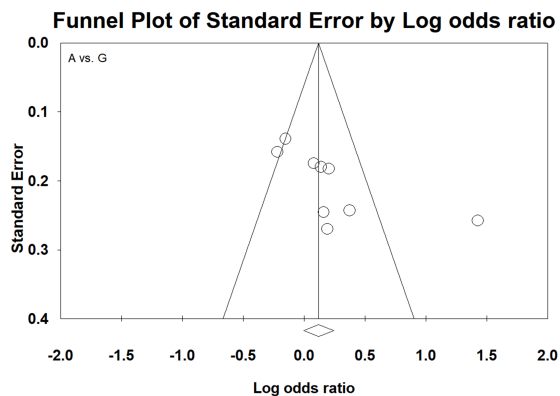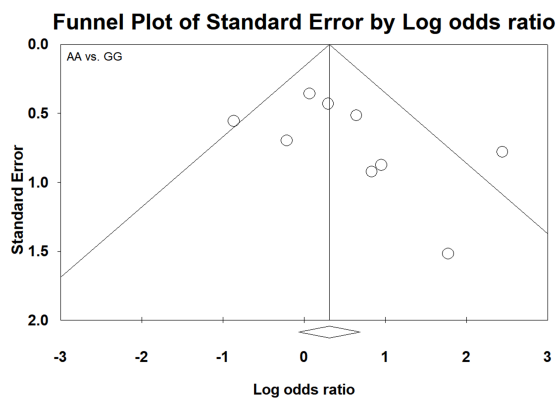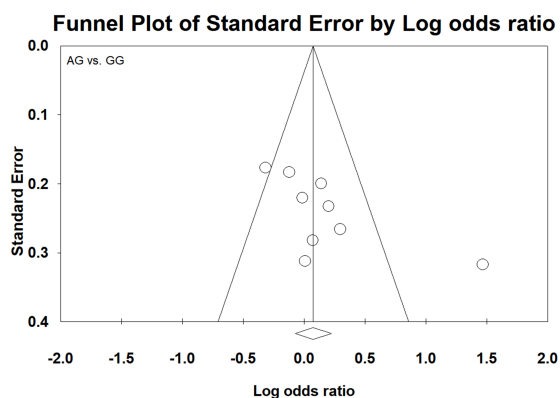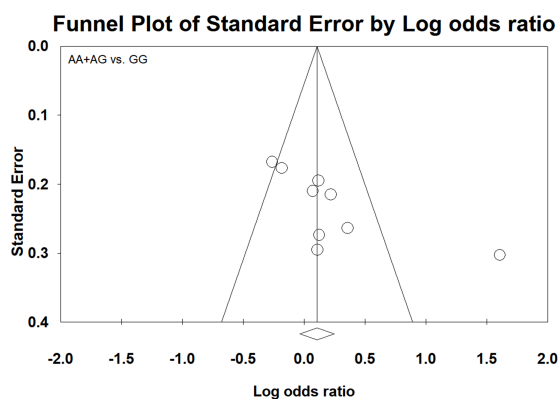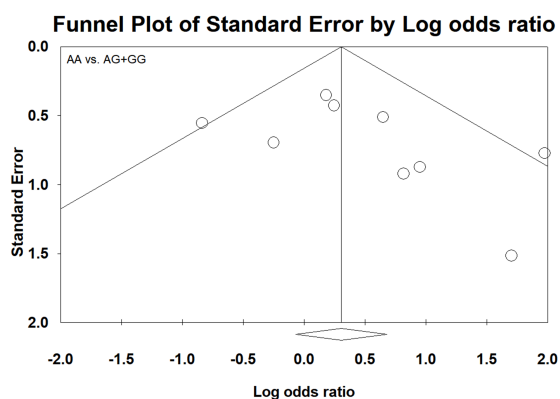

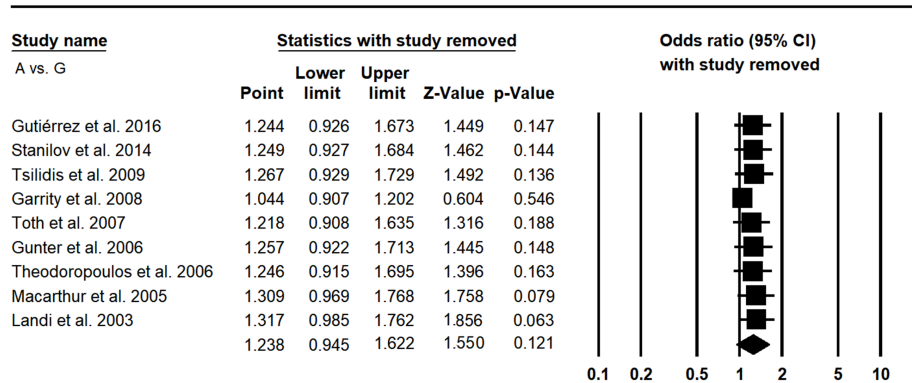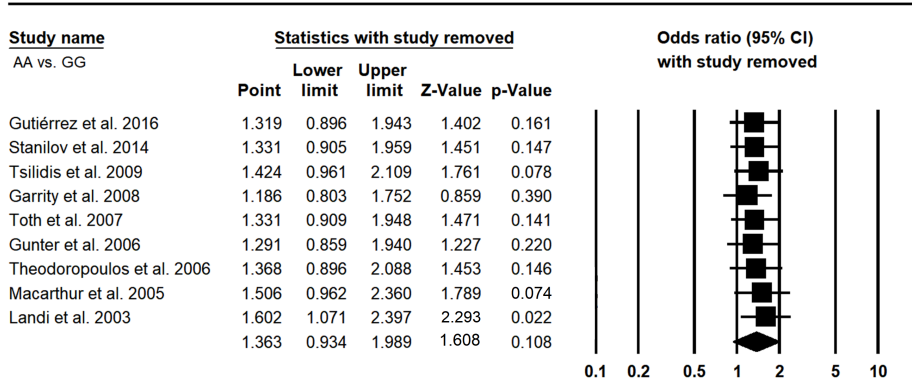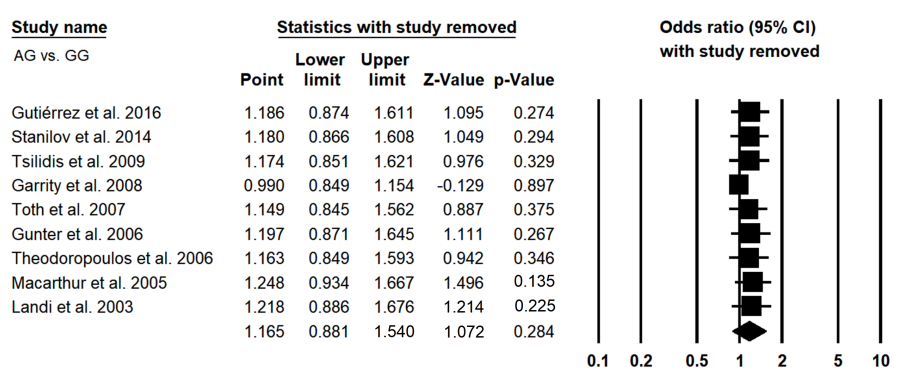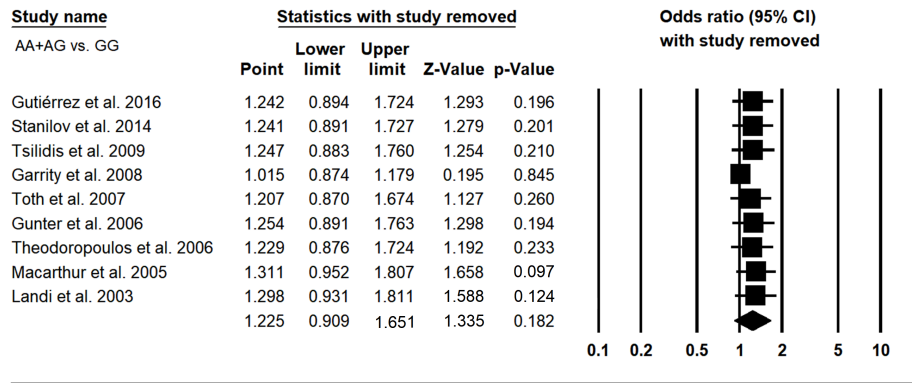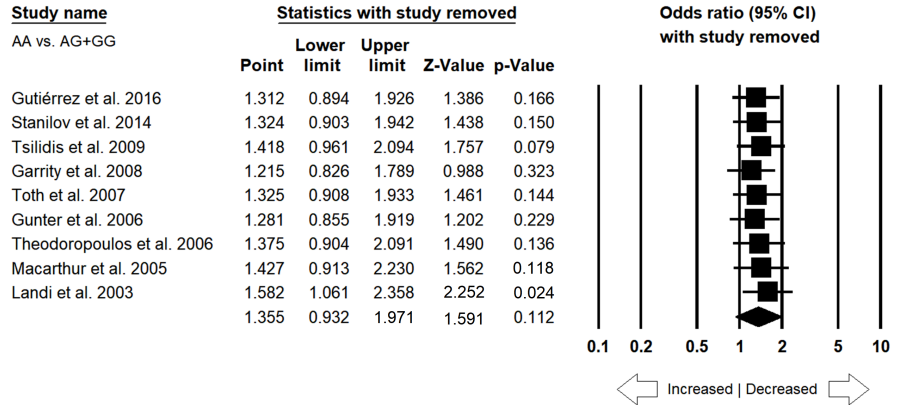

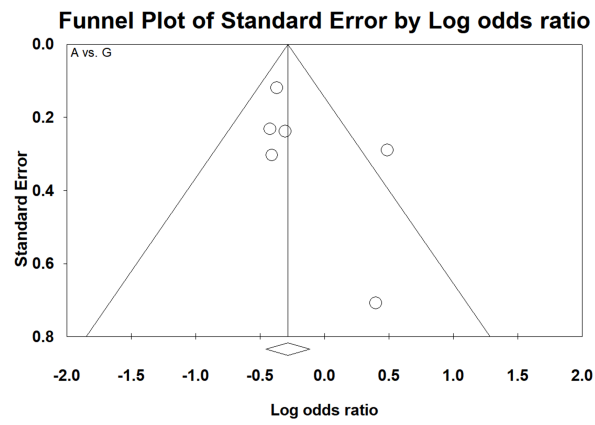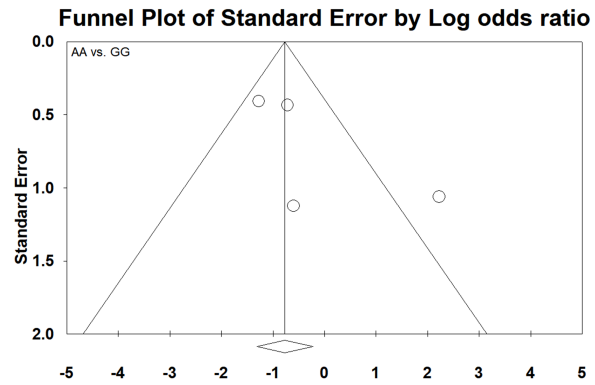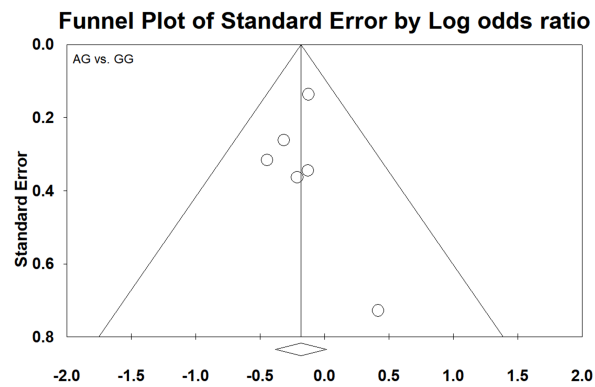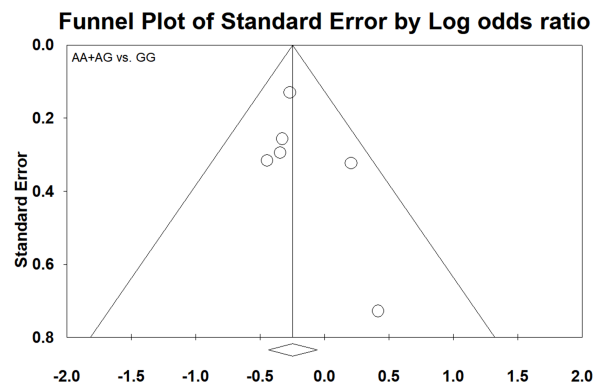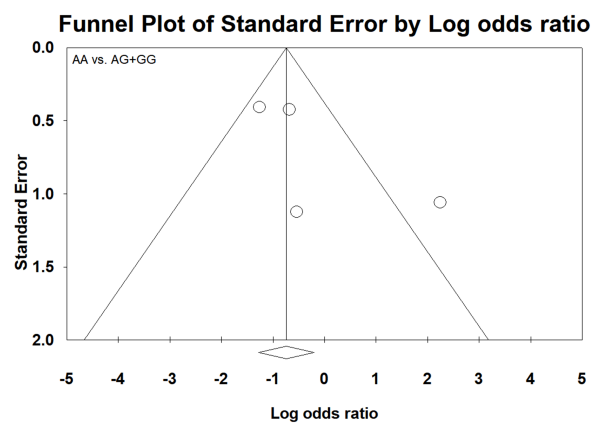

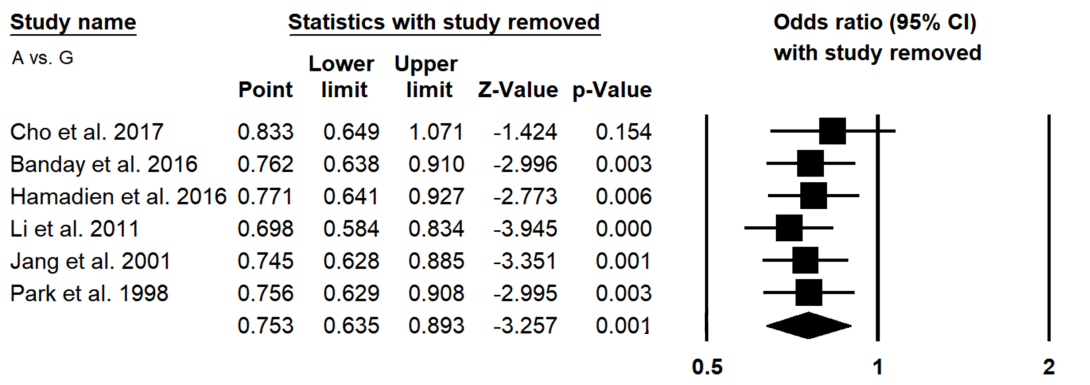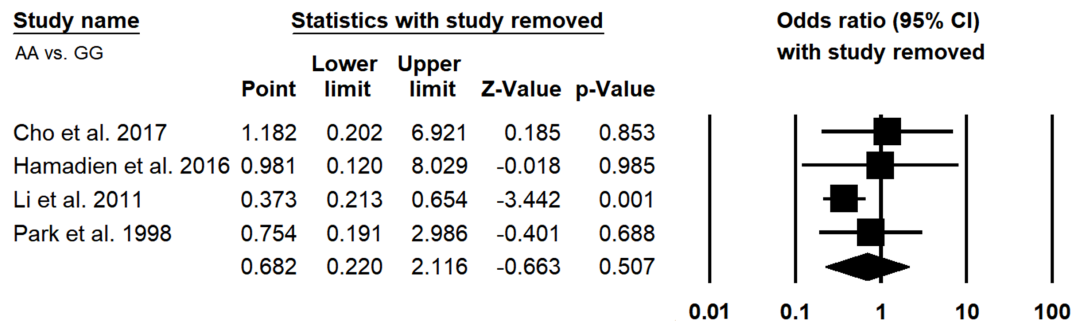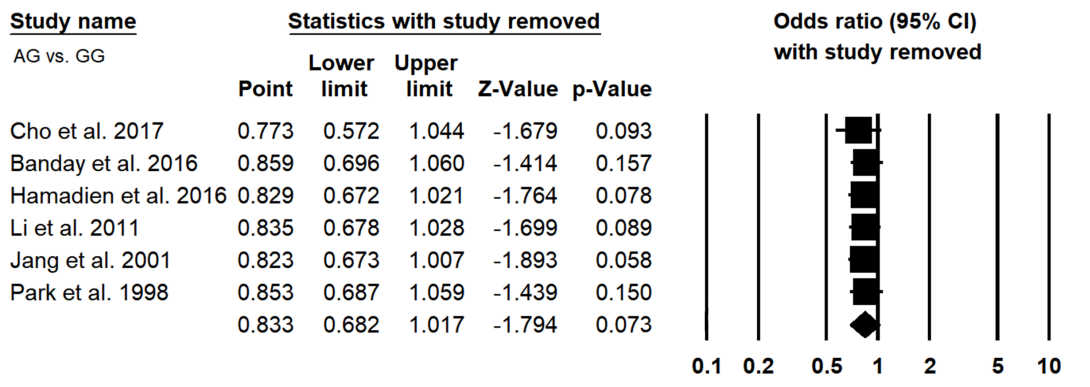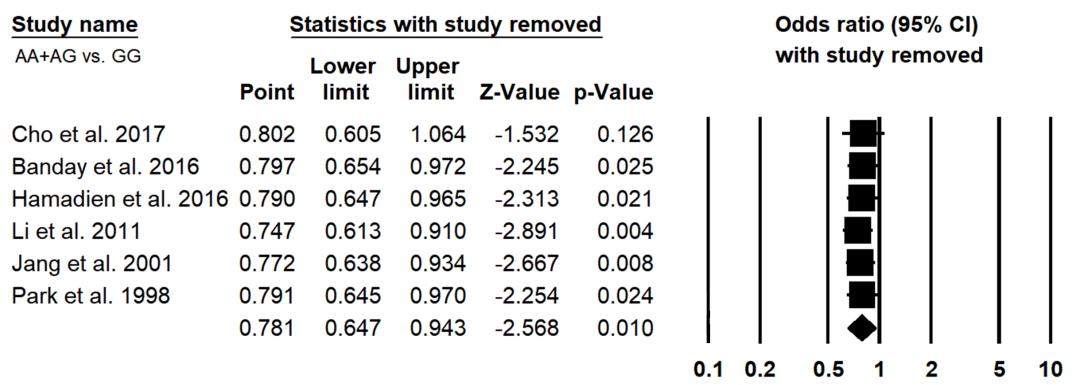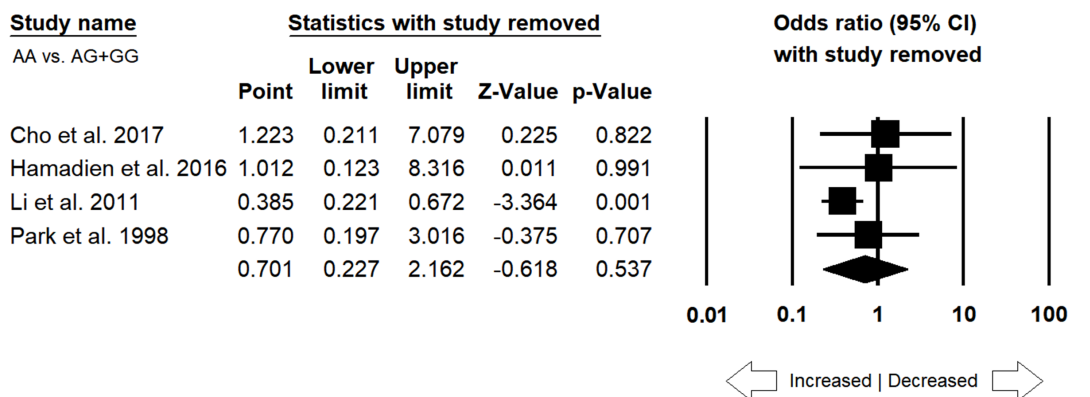

Supplement: Supplementary file 1 [file bsr20181052_Supp1.pdf]
